# Conical Microstructure Flexible High-Sensitivity Sensing Unit Adopting Chemical Corrosion

**DOI:** 10.3390/s20164613

**Published:** 2020-08-17

**Authors:** Yangyang Wang, Jiangyu Deng, Junping Duan, Binzhen Zhang

**Affiliations:** 1 Key Laboratory of Instrumentation Science & Dynamic Measurement, North University of China, Ministry of Education, Taiyuan 030051, China; S1806149@st.nuc.edu.cn (Y.W.); S1706094@st.nuc.edu.cn (J.D.); duanjunping@nuc.edu.cn (J.D.); 2 School of Instrument and Electronics, North University of China, Taiyuan 030051, China

**Keywords:** chemical corrosion, flexible electronics, cone microstructure, motion detection

## Abstract

Sensor technology is one of the three pillars of information technology. This paper aims to discuss the problems of insensitive detection, poor stability, and uncomfortable wearing of sensors in the fields of human–computer interaction, 5G communication, and medical detection. A sensing unit with a microstructured flexible sensing front end is a cone-like structure with a single size of 18–22 μm. They are evenly distributed and can reach 2500 units per square millimeter. In the pressure range, the sensitivity of the sensor unit is 0.6 KPa^−1^ (no microstructure sensitivity at 0.15 KPa^−1^), and the response time is fast (<600 ms). After 400 repeated stretching experiments, the sensor unit can still maintain a stable output signal. Due to its flexible characteristics (50% tensile conductivity), the sensor unit can act on human skin and other curved surfaces. According to the prepared sensing unit, good test results can be obtained on the testing of mechanical devices, curved surfaces of human bodies, and non-contact methods. It is observed that the flexible sensor can be applied to various test occasions, and the manufacturing process of the sensing unit will provide new ideas and methods for the preparation of the flexible sensor technology.

## 1. Introduction

In recent years, various sensor devices have emerged endlessly [1,2,3]. As the source technology of information, sensor technology is the foundation and symbol of modern scientific development and has been widely applied to various disciplines and fields. Sensors can convert various physical signals into electrical signals, thereby facilitating data processing and efficient transmission and becoming an important link in communication [4].

With the continuous development of sensor technology, wearable devices begin to appear and gradually play an increasingly important role in people’s lives. The progress of the Internet is promoting the innovation of wearable devices at an alarming rate [5]. The development of wearable devices has put forward a great demand for flexible electronic technology. Because of its softness, stretch ability, and other characteristics, the wearable device has a wide range of applications [6,7,8,9]. A flexible pressure sensor is a kind of flexible device that can be attached to various regular and irregular surfaces to sense surface forces and their distribution. It has a broad application prospect in biomedical and electronic skin devices [10,11,12]. The main requirements of these equipment are durability and small size, which can be bent, stretched, compressed, twisted, and other deformations, and maintain good reliability and integration.

In a general way, the response of flexible pressure sensors can be divided into capacitive, piezoresistive, photoelectric, hall-type, etc. [13,14,15,16,17,18]. Compared with other sensing methods, capacitive sensors have the advantage of low power consumption, small peripheral volume, high stability, etc. These advantages make it an ideal sensor for healthcare and wearable electronics. For a capacitive pressure sensor, the capacitance value changes with the deformation of the flexible dielectric layer between layers, its sensitivity usually depends on the flexible dielectric layer between the layers of flexible microstructure and surface microstructure template-making process [19,20,21,22], and it contains an artificial template (template silicon etching, corrosion solution) and natural biological templates (rose petals [23], lotus leaf surface [24]).

Bao developed a capacitive pressure sensor with high sensitivity (0.55 KPa^−1^) by microstructuring the dielectric layer in 2010 [25]. Compared with the capacitive sensor without microstructure and linear structure, the response speed and sensitivity of the sensor with a pyramid microstructure were significantly higher, and the response to small external pressure was better. Nguyen Thanh Tien prepared a new flexible sensor that can be attached to human skin in 2014, which can simultaneously detect temperature and pressure, achieving a breakthrough in the miniaturization of wearable devices [26]. Canan Dagdeviren also proposed an array pressure sensor structure (0.005 KPa^−1^) in 2014 [27]. Its preparation process is low-cost and suitable for large-scale preparation, achieving a breakthrough in the application of similar devices. With the development of wearable devices, the demand for flexible mechanics sensors has increased greatly, and flexible mechanics sensors have gradually become the focus of academic research. In 2016, Zhang modeled the surface microstructure of lotus leaf and produced a capacitive tactile sensor with a microstructure conductive electrode and polystyrene spheres (PS) microsphere dielectric layer, with a high sensitivity of 0.815 KPa^−1^ [28]. The biomimetic microstructure of the electrode plays an important role in improving the sensitivity and repeatability of the sensor. Zheng proposed an automatic measurement method of the foot arch index based on the flexible membrane pressure sensor in 2020 [29], and the plantar pressure distribution data obtained by the flexible membrane pressure sensor were converted into digital images. The eight-neighborhood correlation pixel method was proposed to remove the interference of isolated noise points, and the row element association algorithm was proposed.

In a highly sensitive flexible pressure sensor device, micron or nanoscale surface structures have proved to be a very simple and effective method for obtaining high sensitivity, especially pyramidal microstructures [30]. A detailed comparison of the pyramidal structure can be found in the Table S1. From the perspective of mechanics, the pyramid microarray surface makes it easier for them to be compressed under the action of external pressure, because the tip structure on the top of the pyramid is easier to be deformed under the force. This effect is manifested as the change of capacitance in capacitance sensing, while the change of resistance in contact sensing. So this special surface construction method is very effective in both capacitive and contact sensing devices.

Herein, the flexible pressure sensor that was based on polydimethylsiloxane (PDMS) and prepared by chemical corrosion technology has the dielectric layer with a double-sided microstructure. Compared with the deep silicon etching technology, the chemical corrosion method has a higher density of microstructures per unit area and a tighter structure. Compared with the biological mold method, chemical corrosion is easier to control the yield and reduces uncontrollable factors. Therefore, a conical microstructure and a flexible dielectric layer are successful prepared. The sensitivity and reliability of the microstructure of organosilicon compounds were produced through packaging and testing. Increasing sensitivity and shortening response time have great potential in various fields, such as wearable electronic skin. This study elucidated that under the premise of a common use of materials, the idea of processing by corrosion method opened up a new processing way for the structure design of highly sensitive flexible sensor with recyclable utilization and low detection.

## 2. Theoretical Research and Structural Design

### 2.1. The Definition of Capacitive Sensor

The flexible pressure sensing unit with microstructure has a high sensitivity response and is skin-friendly and suitable for complex surfaces to external pressure, which has become a research focus of scholars. In recent years, many scholars have been working together to develop sensors with good performance. It has been found in a variety of sensor structure studies that the capacitive pressure sensor structure has become the first choice of many researchers due to its advantages of high sensitivity, high dynamic response, high temperature stability, small environmental adaptability, and simple structure. Therefore, this subject is also based on the theoretical basis of capacitive pressure sensors.

**Figure 1:**
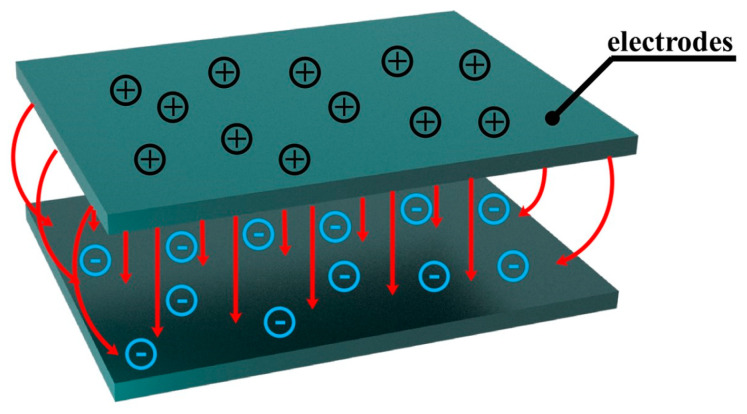
Parallel plate capacitor.

A capacitive-type pressure transducer is a sensor device that takes advantage of external changes to influence the capacitance between the sensing units and converts the measured pressure into a charge output mode corresponding to one of them. As shown in Figure 1, the working mechanism of the structural unit is that the external pressure on the sensing structure changes the pole spacing between the capacitor plates, and the change of the pole spacing will change the capacitance between the two plates. When no other material is used as the dielectric layer between the two plates, the air or vacuum between the plates is used as the dielectric layer.

Without regard to the edge effects, the expression of parallel plate capacitor is:

(1) $$C_{0}=\frac{\varepsilon A}{d}=\frac{\varepsilon_{0}\varepsilon_{r}A}{d}$$

where $\varepsilon_{r}$ and $\varepsilon_{0}$ are the relative permittivity, and in vacuum, d is the distance between two parallel electrode plates, and the air is the medium ($\varepsilon_{r}$ = 1).

If the distance between the electrode plates changes by Δd, the capacitance change is ΔC. By making a systematic theory analysis, the sensitivity of the capacitive sensing unit is the following expression:

(2) $$K=\frac{\Delta C}{C_{0}}/\Delta d=\frac{1}{d}$$

According to the varying parameters, the capacitive sensors are divided into three types, which are variable spacing type, variable area type, and variable dielectric constant type. This subject is a fixed capacitance sensor, it is easiest and most useful to vary its spacing. It can be seen that the smaller the plate spacing is, the space between the pole plates is compressed, the greater the shape variable of the structure is. The sensor unit has higher sensitivity. Therefore, this subject takes the variable spacing sensor as the experimental object for input analysis.

### 2.2. Selection of Flexible Substrates

Based on the above analysis, it is known that the sensitivity of the capacitive pressure sensor can be improved by microstructuring the flexible dielectric layer. The material deforms when the force is exerted, and it quickly returns to its original state when the force is absent. However, different flexible substrates have different sensitivities to the sensors produced, and there are great differences in technology and material properties. 

At present, common flexible materials include polyvinyl alcohol (PVA), polyester (PET), polydimethylsiloxane (PDMS), etc. PDMS compared with other flexible material, it not only convenient, with chemical stability, high permeability, and thermal stability but also overcomes the manufacturing difficulties, such as high cost. Therefore, PDMS will be selected as the flexible substrate for further research.

### 2.3. Selection of the Microstructure

COMSOL Multiphysics software is used to analyze the force of the geometric model of the flexible substrate. Models of dielectric layer substrates with hemispheres, cubes, cuboids, tri-pyramid, cone microstructure, and non-microstructure were established, respectively. The same point charge is applied to the flexible substrate for mechanical simulation (F = 0.0001 N). Table 1 shows the shape variables at the center point obtained by simulation.

In the above table, it can be known that the maximum deformation of the electrodes with different microstructures under pressure is different. Various electrodes also have different sensitivity. In the simulation structure, the shape variables of tri-pyramid and cone are the largest. Therefore, the cone structure is determined as the dielectric layer.

## 3. Preparation

The tightly arranged cone array of microstructures will be vertically compressed and laterally extended during stress and recovery and will undergo periodic changes during testing. Within a certain limit, compression, bending, torsion, etc., will not destroy the structure itself. It shows good repeatability and is a potential bionic human skin fold structure.

### 3.1. Fabrication of Dielectric Layer Cone Microstructure

The cone structure template was fabricated by chemical etching silicon wafer. The microstructural substrate for the dielectric layer of the flexible sensor was obtained by multiple PDMS die flipping. The flexible dielectric layer is microstructured on both upper and lower surfaces, and the cone-like microstructural template can be used repeatedly and recycled, which is beneficial to the mass production of the flexible dielectric layer and further reduces the production cost.

**Figure 2:**
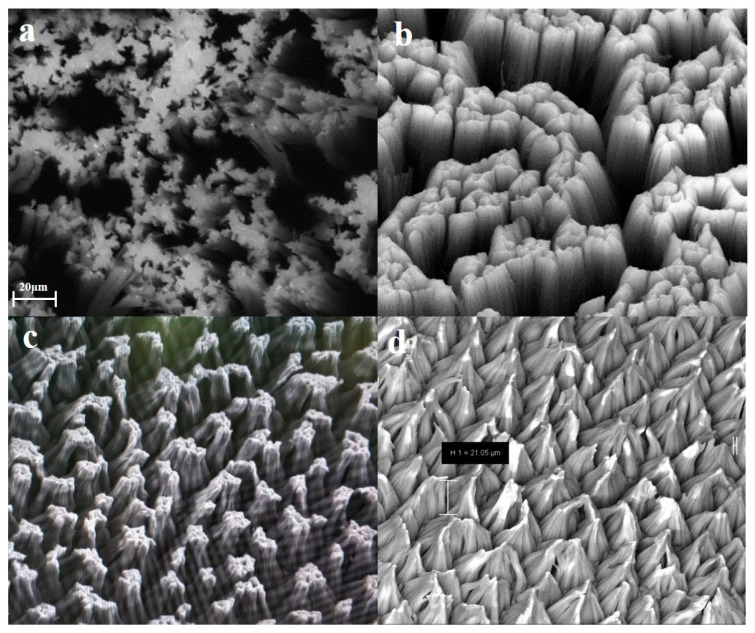
Scanning electron microscopy(SEM)diagram of the silicon wafer corroded in different time periods. (**a**) 15 min, (**b**) 1 h, (**c**) 2 h, and (**d**) 3 h.

After the silicon wafer was cleaned, it was baked at 150 °C through Hexamethyl Disilazane (HMDS) vacuum oven (PVD-090-HMDS) to break the -OH group in the silanol group and eliminate the residual water molecules on the surface. Then, we placed the processed silicon wafer in the mixed solution of AgNO_3_ and HF for 90 s to deposit a certain concentration of silver nanoparticles on the surface of the silicon wafer, and then, the silicon wafer in H_2_O_2_ and HF solution under the environment of Ag nanoparticles as a catalyst chemical corrosion was carried out for 15 min, 1 h, 2 h, and 3 h, respectively. The electron microscope effect diagram is shown in Figure 2. 

**Figure 3:**
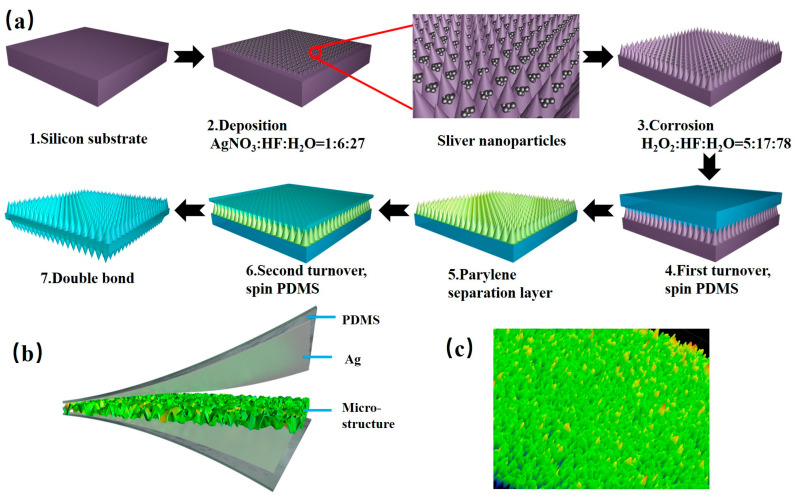
Fabrication process of the dielectric layer. (**a**) Main processes of microstructure manufacture, deposition, corrosion, first turnover, and second turnover. (**b**) Illustration of the capacitive pressure sensor based on a microstructured electrode. (**c**) Characterized 3D microstructure surfaces under laser scanning microscope (LSM). PDMS—polydimethylsiloxane.

Based on the silicon wafer corrosion for 3 h, a microstructure with a well-regulated cone shape and a compact arrangement are obtained. Each microstructure has a high degree of similarity, and its microstructure size is about 25 μm high, which is very suitable for the microstructure of the sensor in. After taking out the above microstructured silicon wafer, we cleaned it and span-coated it with PDMS (200 μm thick) on the surface of the silicon-based cone array structure. After demolding, a PDMS sub-mold substrate with an inverted cone structure was obtained. Next, we used the substrate to invert the microstructure of the cone for the second time, performed easy demolding treatment, and deposited 0.2 μm Parylene as a barrier layer using a parylene vacuum vapor deposition coating instrument (PDS2010), which was beneficial to the separation of the secondary mold matrix and the microstructure matrix. The PDMS film is peeled off to obtain a PDMS sub-mold substrate with an inverted cone structure. Finally, the smooth surface of the PDMS substrate with a single-sided cone structure was smeared with a mixture of PDMS and curing agent in the ratio of 10:1. Bonding with the smooth surface of another piece of single-sided cone structure PDMS substrate to obtain a flexible double-sided cone microstructure dielectric layer. The main fabrication process of the cone microstructure of the flexible pressure sensor is shown in Figure 3.

### 3.2. Fabrication and Analysis of Flexible Electrode

#### 3.2.1. Metal Electrode Surface Treatment

First, we treated the PDMS film substrate with oxygen plasma process for 60 s and, then, continued to use the Sodium Dodecyl Sulfate (SDS) chemical modification to achieve the permanent hydrophilic property of the PDMS film material surface. This step can avoid direct displacement or detachment of the metal material and the substrate after stretching. After that, the PDMS flexible substrate was fixed on the magnetron sputtering machine, and a silver film with a thickness of 1 μm was sputtered on the surface. Finally, the flexible electrode was manufactured.

**Figure 4:**
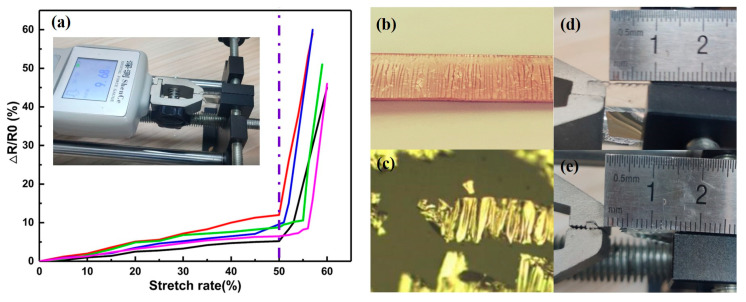
(**a**) Relationship between pull-up rate and resistance change. (**b**) The intuitive structure of the surface of the stretching electrode. (**c**) Microstructure of stretched metal electrode surface. (**d**,**e**) Comparison of the electrode length before and after stretching, 9 mm and 15 mm.

Based on the stretch ability characteristics of the flexible electrode, the electrical performance of the flexible electrode during the flexible stretching process is characterized. In addition, we used the precision resistance tester to record the resistance change value and the deformation relationship during the stretching process and obtained the curve change chart. Figure 4 shows the tensile fracture experiment of the electrode. When the tensile length reaches 150%, the tensile sample is 8 mm long and 20 mm wide, and the silver electrode is 1μm thick. The tensile force of the tensile tester is 9.68 N, and the resistance change rate reaches infinite. The metal on the surface of the electrode will break, cannot conduct, and cannot realize the original function of the electrode. Figure 4b shows the surface metal cracks that can be observed with the naked eye after stretching. Figure 4c shows the peeling and fracture of the metal structure observed under a confocal microscope, which shows that the structure can have good conductivity in the range of less than 50% elongation. The stretch performance of the flexible electrode far exceeds the 10% stretch rate in smart wearable devices.

#### 3.2.2. Electrode Performance Test

**Figure 5:**
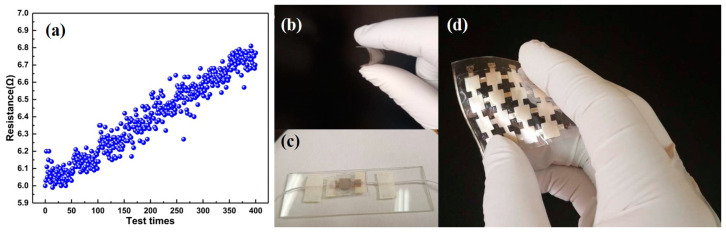
(**a**) 400 repeated tensile tests; (**b**) flexible characteristics of sensing unit; (**c**) physical figure of sensing unit; (**d**) physical drawing of sensor array.

1. Initial resistance test:
  First, the basic performance of the flexible electrode plate was tested. Flexible electrode structures with different diameters were prepared, which were 300, 800, 1200, and 2000 μm respectively, but their lengths were all 1 cm. Take ten groups of diameters for each category and calculate their average. Through the test, the resistance of the electrode was about 6 ± 0.2 Ω. It was proved that the flexible electrode plate made by the above method has very high stability.
2. Repeatability test:
  In order to verify the flexible electrodes in long-term repeated use of the situation, the process of selecting the initial resistance value was 6 Ω flexible electrodes and 400 reciprocating tensile experiments. The simulation device is used to record the electrode resistance value of each stretch back to the initial state at 5% in real time, as shown in Figure 5a. Compared with the initial resistance value, the maximum change after 400 times is 0.8 Ω, and the electrode can remain stable. The performance testing and optimization of each component were carried out, and the final packaging process was completed. The package flexible sensor structure is shown in the Figure 5b–d.

## 4. Measurement of Piezoelectric Sensing Effect of Cone Array Structure

Next, the finished product of the flexible sensor will be tested in multiple experimental environments to explore its test information in biological joints, larynx test information in adult men, different word pronunciation, and other environmental test information. The testing platform mainly includes an impedance analyzer, vertical pressure tester, and other instruments.

### 4.1. Effect Test of Flexible Pressure Sensing Unit

#### 4.1.1. Sensitivity

**Figure 6:**
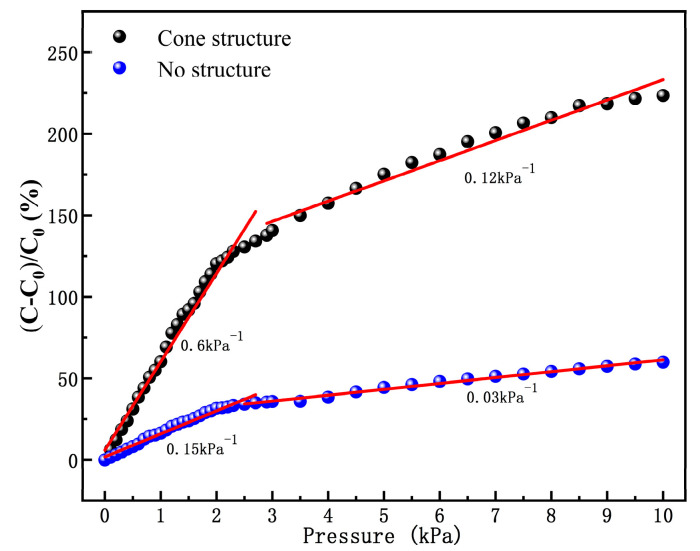
Pressure–response curves for microstructural and non-microstructural.

When the flexible sensor is affected by external conditions and the signal to be tested changes in relative units, the greater the change in the displayed test result, the more intuitively the amount of signal change can be observed. The comparison between the test without a microstructure and the prepared flexible dielectric layer with a microstructure was carried out, and a pressure increasing load test was performed on the sensor units of the two structures. The measured values are shown in Figure 6. Sensitivity is the slope of a straight line drawn at two points.

Analysis of the test results shows that the two types of flexible sensing structures have two linear response regions within the tested range. When the applied pressure load is less than 2 KPa, the sensitivity without microstructure is 0.27 times that of the flexible pressure sensing unit with a microstructure. When the sensor unit is subjected to external pressure, the distance between the tip and the plate of the cone structure decreases rapidly, leading to the increase in capacitance value. In contrast, the sensor unit without microstructure only shows the physical strain effect of extrusion when subjected to external pressure. When the external load pressure exceeds 3 KPa, the cone microstructure sensing unit shows a low sensitivity of 0.075 KPa^−1^, because the middle dielectric layer microstructure shape variable has reached full, and the distance between the two electrodes changes relatively little when the microstructure is squeezed, thus a second sensitivity linear interval appears. By comparison, the sensor units with microstructures in both regions show high sensitivity.

#### 4.1.2. Low Detection and Instantaneous Response Tests

**Figure 7:**
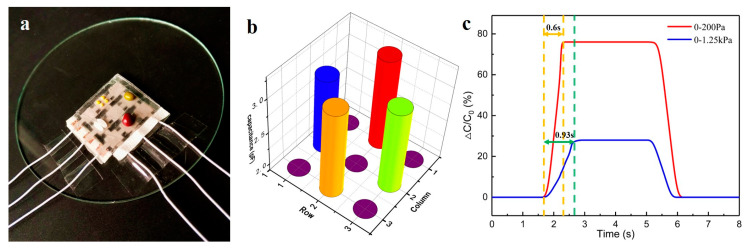
Sensitivity test. (**a**) Low-load sensor array; (**b**) histogram of low detection response; (**c**) instantaneous response test curve.

In order to verify that the flexible sensor unit based on cone microstructure can complete a high-quality inspection report under low load, red bean, mung bean, rice, and millet were placed on the surface of the flexible sensing unit. The data before and after the capacitance conversion were tested, and the low detection response curve of the sensor structure was obtained, as shown in the Figure 7a,b below. In order to increase the reliability of the experiment, the sample was loaded several times, and the test results were the same. The surface pressure contacted by the sensing unit of the tested object in the appeal experiment was about 100 Pa, and it was observed that the instantaneous response time of the sensing unit was less than 1 s when the pressure was low, showing good stability. The response times of two different groups of loads are analyzed below: one is small loads (0–200 Pa), and the other is large loads (0–1.25 KPa). After multiple measurements, we took the average response time and drew the instantaneous response test curve, as shown in Figure 7c. Based on the analysis of the results, when the pressure is loaded and released, the shape variable of the flexible sensor is larger, making it more elastic. This reflects in the instantaneous response, as when the load is loaded with a larger load, it is faster than that when the load is loaded with a smaller load.

### 4.2. Study on the Application of Flexible Pressure Sensing Unit

**Figure 8:**
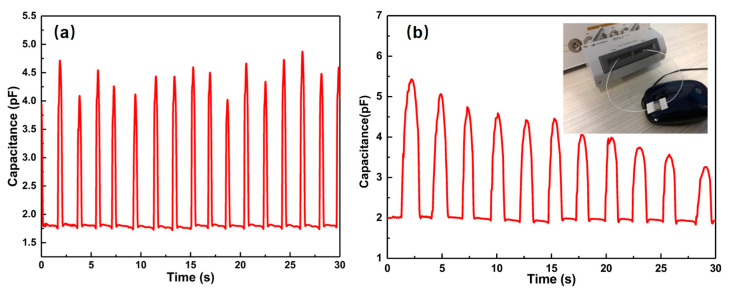
Mechanical mouse test (**a**) quick mouse response; (**b**) normal speed response of mouse.

Stick the prepared flexible pressure sensing unit on the click point of the left mouse button and fingertip. The wire is used to introduce the external signal port of the impedance analyzer, as shown in Figure 8. When giving the mouse a series of press periodic signals, we observed the rule of the signal from the sensor unit periodically change, which illustrates that the flexible pressure sensing unit can accurately test the effect on mouse click force and frequency.

**Figure 9:**
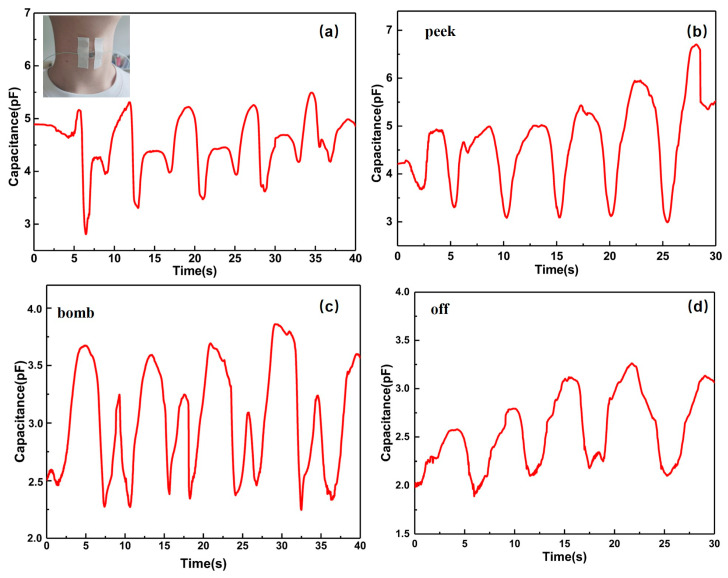
Human laryngeal movement information test (**a**) laryngeal movement test; (**b**) word “peek” test; (**c**) word “bomb” test; (**d**) word “off’ test.

The flexible pressure sensing unit was fitted to the larynx of an adult male. Firstly, the Adam’s apple moved up and down. The signal acquired by the sensor unit followed the up and down movement of the Adam’s apple and presented regular and periodic signal changes, as shown in Figure 9a. The second step was to say the English word “peek” in a normal way. The sensor unit also presented regular and periodic signal changes, as shown in Figure 9b–d. Because the irregular curved shape of the human body’s surface, the sensor unit will be in a slightly curved state when it is attached to the throat. Even in a stable state, a certain pressure is applied to the sensor unit, making the initial capacitance slightly increased. By comparing the data, the results of different types of exercise information show great differences. The results show that the flexible sensing unit can detect the same object in different ways and judge the motion information state of the object. 

**Figure 10:**
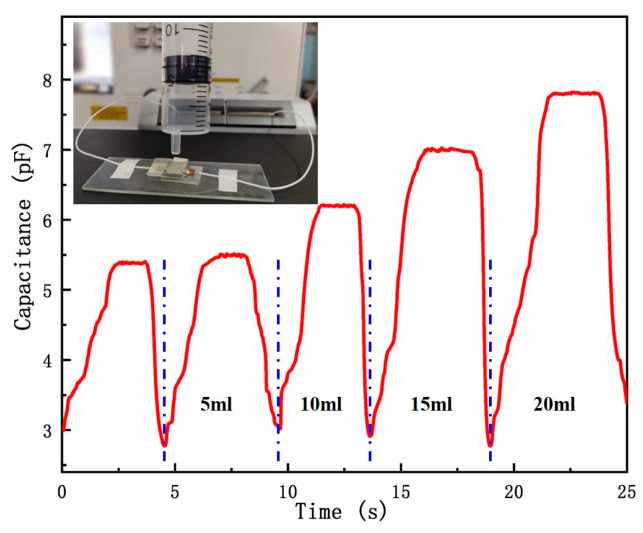
Test of non-contact information by flexible pressure sensing structure.

The flexible pressure sensing unit is characterized by high sensitivity and strong non-contact signal acquisition capability. As shown in Figure 10, the sensor unit was placed in a horizontal state in the experiment. We aligned the sensor unit with a syringe without a needle tip, which was located 1 cm above the sensor unit. Within 0.5 s, the gas flow of 5 ml, 10 ml, 15 ml, 20 ml was sprayed to the sensing unit. The air piston moved up and down, and the syringe was hollowed to be compressed. When the gas was pushed out of the syringe, the curve of capacitance change quickly reached a peak. The signal transmission in the middle was completely carried out with air as the medium. The test results show that the high sensitivity of the flexible sensor unit is used for non-contact information acquisition. It has application prospects in sonic boom detection, decibel detection, and air vibration.

## 5. Conclusions

In summary, we built a highly sensitive flexible pressure sensor based on a cone surface structure inspired by human skin folds. For the PDMS electrode substrate and the dielectric layer, the middle class used a chemical etching method to develop the cone preparation of the dielectric layer and a sensor with high sensitivity (0.6 KPa^−1^), an ultra-low detection limit, good stability in repeated trials (400 times), and fast response. In addition, the process method and process to solve the production cost is high, which is the problem of mass preparation. Because of the flexibility of the PDMS’s unique performance and for the sensor to achieve the random touch of the finger click of the human body recognition, these remarkable characteristics show that the developed sensor can be more widely used for responding to tiny pressure loads (e.g., intelligent robots, artificial skin, health monitoring, and human movement monitoring). Therefore, we think that the flexible sensors in smart wearable electronics have broad prospects for applications in artificial intelligence, and we expect it to be applied to the next generation of advanced electronic equipment.

## Figures and Tables

**Table 1:** Maximum shape variables of different microstructures.

Microstructural	Hemisphere	Cube	Cuboid	Tri-Pyramid	Circular Cone	No Microstructure
Size (μm)	R = 20	a = 20	A = 20	h = 20	H = 20	--
Maximum Shape Variable (μm)	71.874	70.564	70.392	72.513	72.924	67.352

## Supplementary Materials

The following are available online at https://www.mdpi.com/1424-8220/20/16/4613/s1.
