# Supplementary material for: Conical Microstructure Flexible High-Sensitivity Sensing Unit Adopting Chemical Corrosion
Source: Sensors (Basel). 2020 Aug 17;20(16):4613. doi: 10.3390/s20164613 (PMC7472029; doi:10.3390/s20164613)
Supplement: Supplementary file 1 [file sensors-20-04613-s001.pdf]

sensors

MDPI

Letter

Conical Microstructure Flexible High-Sensitivity Sensing Unit Adopting Chemical Corrosion

Yangyang Wang [1,2], Jiangyu Deng [1,2], Junping Duan [1,2] and Binzhen Zhang [1,2,*]

1 Key Laboratory of Instrumentation Science & Dynamic Measurement, North University of China, Ministry of Education, Taiyuan 030051, China; S1806149@st.nuc.edu.cn (Y.W.); S1706094@st.nuc.edu.cn (J.D.); duanjunping@nuc.edu.cn (J.D.)

2 School of Instrument and Electronics, North University of China, Taiyuan 030051, China

* Correspondence: zhangbinzhen@nuc.edu.cn; Tel.: +86-139-3422-1669 (B.Z.)

Few articles have been written about the use of chemical corrosion and cone microstructure. Therefore, we found some articles that etched the microstructure of the pyramid and counted the density of their patterns.

Table A1. Contrast of pattern density and sensitivity of etched microstructures.

Microstructure Width/Height/Spacing (μ m)	Manufacture Method	Pattern Density	Materials	Sensitivity	Ref.
50/60/41	Silicon etching	30.19%	PDMS	2 Kpa^{-1}	1
6/3/1.33	Silicon etching	67%	PDMS	8.2 Kpa^{-1}	2
6 /6 /6	Silicon etching	25%	PDMS	0.55 Kpa^{-1}	3
6 /3 / 3.8	Silicon etching	37.48%	PDMS	0.58 Kpa^{-1}	4
8 /4 /8	Silicon etching	25%	PSS/PUD	4.88 Kpa^{-1}	5
4.5/4.5/2	Wet etching	47.93%	PDMS/GO	5.5 Kpa^{-1}	6
4 /2 /5.6	Wet etching	14.24%	PGS	0.76 Kpa^{-1}	7
10/10/5	Wet etching	44.44%	PDMS	0.31 Kpa^{-1}	8

The microstructure in this manuscript is elongated. The same bottom area, compared with a smaller height, a higher microstructure can provide a larger dynamic range, that is, a larger amount of deformation. The pattern density in this manuscript is close to 100%.

References

1. Ho-Hsiu, C.; Amanda, N.; Alex, C.; John, W. A chameleon-inspired stretchable electronic skin with interactive colour changing controlled by tactile sensing. *Nat Commun*, **2015**, *6*, doi:10.1038/ncomms9011.
2. Schwartz, G.; Tee, B.C.K.; Mei, J.G.; Appleton, A.L.; Kim, D.H.; Wang, H.L.; Bao, Z.N. Flexible polymer transistors with high pressure sensitivity for application in electronic skin and health monitoring. *Nature Commun.* **2013,** *4*, doi:10.1038/ncomms2832.
3. Mannsfeld, S.C.B.; Tee, B.C.K.; Stoltenberg, R.M.; Chen, C.; Barman, S.; Muir, B.V.O.; Sokolov, A.N.; Reese, C.; Bao, Z.N. Highly sensitive flexible pressure sensors with microstructured rubber dielectric layers. *Nat. Mater.* **2010**, *9*, 859–864, doi:10.1038/nmat2834.
4. Pang, C.; Koo, J.H.; Nguyen, A.; Caves, J.M.; Kim, M.G.; Chortos, A.; Kim, K.; Wang, P.J.; Tok, J.B.H.; Bao, Z.A. Highly Skin-Conformal Microhairy Sensor for Pulse Signal Amplification. *Adv. Mater.* **2015**, *27*, 634–640, doi:10.1002/adma.201403807.
5. Choong, C.L.; Shim, M.B.; Lee, B.S.; Jeon, S.; Ko, D.S.; Kang, T.H.; Bae, J.; Lee, S.H.; Byun, K.E.; Im, J., et al. Highly Stretchable Resistive Pressure Sensors Using a Conductive Elastomeric Composite on a Micropyramid Array. *Adv. Mater.* **2014,** *26*, 3451–3458, doi:10.1002/adma.201305182.
6. Zhu, B.W.; Niu, Z.Q.; Wang, H.; Leow, W.R.; Wang, H.; Li, Y.G.; Zheng, L.Y.; Wei, J.; Huo, F.W.; Chen, X.D. Microstructured Graphene Arrays for Highly Sensitive Flexible Tactile Sensors. *Small* **2014**, *10*, 3625–3631, doi:10.1002/smll.201401207.

7. Boutry, C.M.; Nguyen, A.; Lawal, Q.O.; Chortos, A.; Rondeau-Gagne, S.; Bao, Z.N. A Sensitive and Biodegradable Pressure Sensor Array for Cardiovascular Monitoring. *Adv. Mater.* **2015**, *27*, 6954–+, doi:10.1002/adma.201502535.
8. Lin, L.; Xie, Y.N.; Wang, S.H.; Wu, W.Z.; Niu, S.M.; Wen, X.N.; Wang, Z.L. Triboelectric Active Sensor Array for Self-Powered Static and Dynamic Pressure Detection and Tactile Imaging. *ACS Nano* **2013**, *7*, 8266–8274, doi:10.1021/nn4037514.
